# Efficacy and Safety of Baclofen 40 mg/20 mL in an Intrathecal Infusion System

**DOI:** 10.3390/life16030473

**Published:** 2026-03-14

**Authors:** Riccardo Marvulli, Serena Gervasi, Giuseppa Lagioia, Lucrezia Dell’Olio, Serena Caforio Montesardo, Marisa Megna, Maurizio Ranieri

**Affiliations:** Department of Translational Biomedicine and Neuroscience (DiBraiN), University of Bari Aldo Moro, G. Cesare Place 11, 70125 Bari, Italy; serena.gervasi@yahoo.it (S.G.); giusy.lagioia@yahoo.com (G.L.); lucreziadellolio20@gmail.com (L.D.); caforio.serena@gmail.com (S.C.M.); marisa.megna@uniba.it (M.M.); maurizio.ranieri@uniba.it (M.R.)

**Keywords:** baclofen, neteka, intrathecal therapy, spasticity, ITB, safety, efficacy, UMS, cerebral palsy

## Abstract

Background: Spasticity is a common feature of Upper Motoneuron Syndrome and is frequently treated with intrathecal baclofen (ITB) when oral therapy is ineffective or poorly tolerated. Different baclofen formulations are available for intrathecal use, but evidence regarding their clinical equivalence in patients requiring high daily doses remains limited. Methods: Thirty adult patients (mean age 37 ± 5.8 years) receiving long-term ITB therapy were switched from four vials of baclofen 10 mg/5 mL to a single vial of baclofen 40 mg/20 mL, while maintaining the same total drug amount and reservoir concentration. Patients were divided into two groups: in 15 patients, the daily baclofen dose was maintained unchanged, while in the remaining 15 patients a 10% dose reduction was attempted. Clinical outcomes were assessed at baseline and during follow-up using validated scales for spasticity, pain, and functional status. Results: In patients with unchanged daily dosing, no clinically relevant differences were observed in spasticity, pain, or functional independence after switching formulation, supporting clinical equivalence between the two preparations. Conversely, the 10% dose reduction proved clinically unsustainable in patients requiring high daily doses and required restoration of baseline dosing in all cases. No adverse events or formulation-related complications were observed. Conclusions: Switching to baclofen 40 mg/20 mL is safe and clinically equivalent to the conventional 10 mg/5 mL formulation when the daily dose is maintained unchanged. Dose reduction appears unsuitable in patients requiring high daily intrathecal baclofen doses.

## 1. Introduction

Spasticity is one of the main components of Upper Motoneuron Syndrome (UMS). UMS is characterized by a combination of positive and negative signs that emerge in patients following cerebral palsy, stroke, multiple sclerosis, brain injury, spinal cord injury, and other lesions of the central nervous system [1]. Overall, patients may experience a combination of negative signs, such as muscle weakness and loss of dexterity, and positive signs, including muscle overactivity, hyperactive tendon reflexes, clonus, and flexor spasms. Therefore, dysfunction involving both pyramidal and extrapyramidal pathways, with a predominant role of the latter, has been proposed as a potential explanation for the complex pathophysiology of UMS [2].

Although spasticity due to spinal and cerebral lesions has rarely been directly compared, no qualitative differences in clinical presentation appear to exist. Nevertheless, quantitative differences may occur, as spastic signs are generally less pronounced in cerebral lesions compared with spinal lesions. This observation has been attributed to the presence of a small number of uncrossed corticospinal tract fibers supplying the affected side in cases of unilateral brain damage [3]. Regardless of the underlying cause, spasticity results from reduced gamma-aminobutyric acid (GABA)-mediated inhibition of spinal reflexes, which impairs muscle relaxation and leads to excessive muscle contraction during the stretch phase, ultimately resulting in abnormal muscle tone (hypertonicity) [4]. The inhibitory effect on motoneurons is primarily presynaptic, selectively depressing excitatory potentials and reducing both monosynaptic and polysynaptic reflex activity in the spinal cord. Moreover, GABA-B receptor–mediated suppression of motor activity appears to be tonically active [4,5].

The management of spasticity includes several treatment options, such as physiokinetic therapy, systemic oral drug therapy, intrathecal infusion therapy, and local antispastic interventions. Various physiotherapeutic approaches, including active and passive mobilization and muscle stretching, may be recommended to improve residual motor function and to prevent muscle contractures or tendon retractions [1,3]. Local antispastic treatment is often successfully achieved through botulinum toxin injections, which induce temporary paresis of hypertonic muscles, typically lasting between four and six months.

Systemic oral pharmacological therapy includes the administration of different agents, such as clonidine, which has been shown to induce marked inhibition of spinal reflex responses in group II motor neurons in animal models [4]; tizanidine, which acts on α2 receptors in the spinal dorsal horn and inhibits the release of substance P, thereby reducing flexor reflex–mediated activity [5]; and benzodiazepines, which enhance the inhibitory action of GABA-A receptors at both presynaptic and postsynaptic levels. Nevertheless, among available drugs, baclofen appears to exert the most pronounced effect on spasticity and can be administered either orally or intrathecally.

The baclofen molecule has one chiral center and a molecular weight of 213.66 g/mol. It exists as a white, odorless crystalline powder, completely insoluble in chloroform and slightly soluble in water (0.712 mg/mL) and ethanol [6,7]. In 1977, baclofen received approval from the U.S. Food and Drug Administration (FDA) for the treatment of spasticity resulting from brain or spinal cord injury and a range of neurological disorders [8]. Baclofen was originally synthesized by Swiss Ciba-Geigy chemist Heinrich Keberle as a lipophilic analog of GABA, namely β-(4-chlorophenyl)-γ-aminobutyric acid [1]. Initially developed as an antiepileptic agent, preclinical animal studies later revealed its potent muscle-relaxant properties, leading to its clinical use in spasticity.

To better understand the role of baclofen in reducing spasticity, its pharmacodynamic properties must be considered. Baclofen is an agonist of GABA-B receptors, which are G-protein-coupled receptors located on both pre- and postsynaptic neurons in the central and peripheral nervous systems [9]. Presynaptically, baclofen reduces calcium influx, thereby impairing neurotransmitter release [10], while postsynaptically, it induces potassium efflux and neuronal hyperpolarization, producing an inhibitory effect [11]. Consequently, baclofen acts through both presynaptic inhibition of neurotransmitter release and postsynaptic suppression of neuronal excitability.

Oral administration of baclofen is typically initiated at a dose of 5 mg three times daily and may be increased up to a total of 80–100 mg/day in adult outpatients. In pediatric patients, an initial daily dose of 0.3 mg/kg is recommended, divided into two to four administrations, with gradual titration up to 2 mg/kg/day, and not exceeding a maximum total daily dose of 60 mg. However, the pharmacokinetic profile of baclofen, characterized by limited lipid solubility and poor penetration of the blood–brain barrier, represents a significant limitation. Only a small percentage of the administered oral dose reaches the central nervous system; for example, oral baclofen doses of 30–60 mg have been associated with cerebrospinal fluid concentrations ranging from 12 to 96 µg/mL [12].

When patients experience intolerance or an inadequate response to oral therapeutic doses, intrathecal delivery of baclofen is recommended. Intrathecal baclofen is administered directly into the cerebrospinal fluid via a tunneled catheter connected to a pump surgically implanted in the abdominal wall, either subcutaneously or subfascially. The pump can be programmed to deliver the drug continuously or intermittently, depending on clinical requirements [13,14]. Patients considered eligible for intrathecal baclofen therapy must undergo a preliminary screening trial to confirm their responsiveness to treatment [15].

The aim of the present study was to evaluate the safety and clinical equivalence of switching to a baclofen 40 mg/20 mL formulation in patients requiring high daily doses of intrathecal baclofen. The primary outcome was to assess the impact of formulation switch on clinical manifestations of spasticity and pain. The secondary objective was to explore whether a modest dose modification could be tolerated in this population, with particular attention to its potential impact on pump refill intervals.

## 2. Materials and Methods

### 2.1. Participants

All patients were selected among those referred to the Spasticity Clinic of the Spinal Unit at the Bari University Hospital Consortium.

To be eligible for inclusion in the study, patients were required to meet all of the following criteria:

• Male or female sex;
• Age ≥ 18 years;
• Diagnosis of cerebral palsy, stroke, multiple sclerosis, brain injury, or spinal cord injury, or presence of severe spasticity of the upper and/or lower limbs not adequately controlled by therapeutic oral baclofen doses;
• Implantation of a pump with a 20 mL reservoir;
• Reservoir filled with a concentrated baclofen solution at 2000 µg/mL;
• Ongoing intrathecal baclofen therapy with a daily dose ≥ 300 µg/day;
• Stable intrathecal baclofen dosing for at least six months prior to enrollment.

Exclusion criteria included age < 18 years, inpatient status, unstable clinical conditions, intrathecal baclofen daily doses < 300 µg/day, or use of baclofen solutions with a reservoir concentration lower than 2000 µg/mL. The latter criterion was necessary to ensure comparability between formulations with identical final concentrations.

Among 33 patients followed at our center, 30 met the inclusion criteria and were enrolled in the study. The final cohort consisted of 30 adult patients (mean age 37 ± 5.8 years) with diagnoses of spastic tetraparesis, spastic paraparesis, or multiple sclerosis, all receiving long-term intrathecal baclofen therapy. All patients had previously undergone surgical implantation of a 20 mL reservoir pump (Medtronic SynchroMed II^®^, Minneapolis, MN, USA), with a mean treatment duration of approximately 10 years.

Concomitant local antispastic treatments, such as botulinum toxin injections, were not considered exclusion criteria, as these therapies were administered to the upper limbs, whereas clinical outcomes related to intrathecal baclofen were evaluated in the lower limbs.

### 2.2. Study Design

At enrollment, all patients underwent a switch in baclofen formulation used for pump refill, from four vials of baclofen 10 mg/5 mL to one vial of baclofen 40 mg/20 mL (Neteka^®^, Sintetica SA, Mendrisio, Switzerland). In all cases, the total drug amount and final reservoir concentration (2000 µg/mL) were maintained unchanged. According to the manufacturer’s product information, both formulations are declared stable within the pump reservoir for up to 180 days.

Following formulation switch, patients were divided into two groups:

• Group A (n = 15): daily intrathecal baclofen dose was maintained unchanged.
• Group B (n = 15): a systematic reduction of the daily intrathecal baclofen dose by 10% was attempted, with the aim of evaluating dose tolerability and its potential impact on pump refill intervals.

The mean daily intrathecal baclofen dose at baseline was 501 ± 10.7 µg/day. Drug delivery was performed through a catheter implanted in the lumbar intrathecal space. Pump refill intervals were calculated using the manufacturer’s software to avoid drug depletion.

All patients and caregivers were provided with direct access to qualified medical staff through a 24/7 on-call telephone service throughout the study period.

Data were assessed before formulation change (T0) and after 15 (T1), 30 (T2), 45 (T3), and 60 days (T4). In Group B, an additional evaluation was performed after restoration of the baseline dose following the unsuccessful dose reduction attempt (T5).

### 2.3. Clinical Assessment

Clinical evaluations were performed using standardized and validated scales. Muscle tone was assessed using the Modified Ashworth Scale (MAS) in the hip adductor muscles and femoral biceps. Spasm frequency and severity were evaluated using the Penn Spasm Frequency Scale (PSFS). Functional status was assessed using the Functional Independence Measure (FIM), while pain intensity was evaluated using the Visual Analog Scale (VAS).

Achilles tendon clonus was also assessed using a dedicated clinical scale. All outcome measures were collected at baseline and at subsequent follow-up time points.

### 2.4. Statistical Analysis

Data distribution was assessed using the Shapiro–Wilk test. Continuous variables are presented as mean ± standard deviation (SD). For Group A (dose maintained), repeated-measures analysis of variance (ANOVA) was used to evaluate longitudinal changes across follow-up time points after verification of normality and homogeneity of variances. For Group B (dose reduction attempt), given the ordinal nature of some clinical scales (e.g., MAS and PSFS) and the limited sample size, the Friedman test for repeated measures was applied to assess differences among baseline (T0), dose reduction phase (T1), and post-restoration assessment (T5). When the Friedman test was significant, pairwise comparisons were performed using the Wilcoxon signed-rank test with Bonferroni correction. A *p*-value < 0.05 was considered statistically significant. Statistical analyses were performed using standard statistical software.

## 3. Results

The study population consisted of 30 adult patients with diagnoses of spastic tetraparesis, spastic paraparesis, or multiple sclerosis, all receiving long-term intrathecal baclofen therapy for a mean duration of approximately 10 years. All patients completed the one-year follow-up period.

### 3.1. Group-Specific Outcomes

#### 3.1.1. Group A—Dose Maintained (n = 15)

For the 15 patients in whom the daily intrathecal baclofen dose was maintained unchanged after formulation switch, clinical stability was preserved throughout the follow-up period. No statistically significant changes were observed in MAS or FIM scores. PSFS, VAS, and Achilles tendon clonus assessments showed stable or mildly improved values compared with baseline (see Table 1).

Pump refill intervals remained consistent with baseline values, and no patient in this group required dose adjustments or unscheduled clinical evaluations.

#### 3.1.2. Group B—Dose Reduction Attempted (n = 15)

In the remaining 15 patients, a systematic reduction of the daily intrathecal baclofen dose by 10% was attempted following the formulation switch. This strategy proved to be clinically unsustainable in all patients in this group.

Dose reduction was associated with worsening of spasticity, increased discomfort, and/or reduced tolerance, leading to restoration of the baseline daily dose in all cases. As a consequence, no clinically meaningful extension of pump refill intervals was achieved in this group. MAS, PSFS, and VAS scores significantly worsened during dose reduction (all *p* < 0.01) and returned to baseline after dose restoration see Table 2).

No serious adverse events occurred during the dose reduction attempt; however, due to the lack of clinical tolerability, the reduced dosing regimen was discontinued.

### 3.2. Safety

Throughout the one-year follow-up period, no episodes of baclofen withdrawal, overdose, infectious complications, or device-related malfunctions were recorded. None of the patients or caregivers required use of the 24/7 on-call medical service.

## 4. Discussion

Intrathecal baclofen (ITB) therapy is an established and effective treatment for severe spasticity; however, it remains associated with a non-negligible rate of complications and logistical challenges. Reported complications include mechanical issues, such as catheter malfunction, cerebrospinal fluid leakage, pump failure, or sudden interruption of drug delivery, as well as infectious complications occurring either postoperatively or, less frequently, following refill procedures. These events have been reported more frequently in pediatric populations than in adults [16].

Abrupt interruption of intrathecal baclofen delivery represents a potentially life-threatening condition and may manifest with severe withdrawal symptoms, including seizures, coma, rhabdomyolysis, disseminated intravascular coagulation, and multisystem organ failure. Conversely, baclofen toxicity may present with muscular hypotonia, nausea, vomiting, hypotension, dizziness, and respiratory depression. In both scenarios, prompt recognition and appropriate management are crucial, as no specific antidote for baclofen overdose is currently available [17].

Although these complications are relatively uncommon, patients may experience end-of-dose effects, typically characterized by increased spasticity in the days preceding scheduled pump refills. Such effects are often more evident to caregivers and physiotherapists and may significantly affect daily care activities and rehabilitation outcomes. For this reason, strategies aimed at simplifying refill procedures and potentially reducing refill frequency are of considerable clinical interest.

In clinical practice, pump refill procedures require highly trained personnel, strict sterile conditions, and adequate infrastructure. Moreover, patients with severe motor impairment often face substantial logistical challenges in accessing specialized centers, including the need for dedicated transportation and caregiver assistance. As ITB therapy is generally lifelong, reducing the procedural burden associated with repeated refills represents an important goal for improving patient and caregiver quality of life.

In addition to its antispastic effects, baclofen also contributes to pain management. The drug modulates pain perception through several mechanisms, including suppression of neuropathic pain, downregulation of sympathetic activity, and reduction in pain associated with muscle spasms [18]. Herman et al. demonstrated that tonic GABAergic activity in the dorsal horn is reduced in neuropathic pain states and that intrathecal baclofen may help restore inhibitory function [19]. Furthermore, baclofen has been shown to inhibit substance P release from presynaptic terminals in laminae I–II of rat spinal cord slices, as assessed by neurokinin-1 receptor internalization [20,21], and to exert supraspinal effects by depressing ascending adrenergic and dopaminergic pathways while facilitating descending noradrenergic pathways [19]. Clinically relevant effects on the reduction in sympathetic nervous system hyperactivity associated with severe brain injury have also been reported [22]. Despite these effects, combination therapy is often required to avoid overdose and potential adverse effects.

The present study was designed to evaluate the safety and clinical equivalence of switching from a conventional baclofen 10 mg/5 mL formulation to a single-vial baclofen 40 mg/20 mL formulation (Neteka^®^) in patients requiring high daily doses of intrathecal baclofen. Our results demonstrate that maintaining the daily dose unchanged while switching formulation preserves clinical efficacy in terms of spasticity control, pain management, and functional status, with no increase in adverse events or end-of-dose effects [23,24]. These findings support the clinical equivalence of the two formulations when administered at identical concentrations and doses.

In contrast, the systematic attempt to reduce the daily intrathecal baclofen dose by 10% proved to be clinically unsustainable in all patients undergoing dose reduction. This finding suggests that patients requiring high daily doses of intrathecal baclofen may have a limited therapeutic margin, and even modest dose reductions may lead to worsening of spasticity or reduced tolerance [25]. Importantly, this negative result should be interpreted as clinically informative, as it indicates that dose reduction strategies may not be appropriate in this specific patient population.

From a practical standpoint, the use of a single-vial 40 mg/20 mL formulation simplified refill procedures by reducing preparation time and the handling of multiple vials, thereby potentially lowering the risk of contamination and procedural errors [25,26]. Although this aspect was not formally quantified, it represents a relevant consideration in routine clinical practice.

While dose reduction was not feasible in patients with high intrathecal baclofen requirements, the documented long-term stability of the 40 mg/20 mL formulation provides a rationale for future investigations aimed at safely extending pump refill intervals. Such strategies should be explored cautiously and may be more suitable for patients with lower daily dose requirements. Importantly, any attempt to extend refill intervals beyond currently recommended limits should be conducted within controlled clinical protocols and supported by careful monitoring [27].

Several limitations of this study should be acknowledged. The sample size was relatively small, and the study was conducted at a single center. Moreover, refill interval extension was not directly tested beyond standard limits, and economic or quality-of-life outcomes were not formally assessed

Nevertheless, the strengths of this study include its pragmatic design, long-term follow-up, and focus on a clinically relevant population requiring high-dose intrathecal baclofen therapy.

Overall, our findings indicate that switching to baclofen 40 mg/20 mL is safe and clinically equivalent to the conventional formulation in patients with high daily intrathecal baclofen requirements, while systematic dose reduction should be avoided in this population. Future multicenter studies are warranted to further explore whether the increased stability of this formulation can be leveraged to safely optimize refill strategies in selected patient groups [28].

Korhonen et al. [27] recently tested the stability of baclofen in 25 samples taken from 19 patients pump. Each sample had mean baclofen concentration determined with HPLC-DA of 2.002 mg/mL, all of the samples were from the same brand (Baclofen Sintetica, manufacturer: Sintetica SA, Mendrisio, Switzerland). The concentration of the drug did not vary significantly over a mean time of 711 days (SD 135, range 487–976 days) showing that ITB refill intervals could be lengthened to up to one year.

Moreover, according to the medical operators’ involved perspective, the single vial formulation simplifies the refill procedures as it takes less time to fill the syringe and the needle used to fill the syringe has less chance of being contaminated by accidentally hitting the outer part of the vial (knowing that it is only necessary to fill the syringe with one vial instead of four).

Korhonen et al. [27] findings and our findings together provide a basis to further investigate whether, by administering the formulation of baclofen 40 mg/20 mL (Baclofen Sintetica, manufacturer: Sintetica SA, Mendrisio, Switzerland), it might be possible to safely treat patients who need very low daily doses of the drug potentially extending their refill intervals up to one year.

It would be interesting to better investigate this potential outcome, as this change could not only reduce the risk of infection associated with the refill process and improve the quality of life for both patients and carers, but it could also improve the economic management of ITB therapy for the healthcare system.

In fact, shortening the refill time in order to strictly adhere to the stability limit of the drug in the reservoir results in a waste of solution that has to be removed from the ITB system and discarded, as well as an increased amount of sterile equipment used by the medical team. It is necessary to underscore that a multi-centric experience should be prioritized, as the refill time over 180 days on the formulation of baclofen 40 mg/20 mL (Baclofen Sintetica, manufacturer: Sintetica SA, Mendrisio, Switzerland) entails an off-label administration, thereby making it more challenging to enroll patients in a clinical trial.

Furthermore, if that will ever be the case, we would also strongly recommend that a clinical evaluation must be conducted at the midpoint of the refill interval and that a telemedicine evaluation must be conducted at each quarter point, in order to ensure the patient is adequately followed up. A potential limitation of the present study is that concomitant botulinum toxin injections were not considered an exclusion criterion. Although in our cohort botulinum toxin was administered exclusively to the upper limbs, while clinical outcomes related to intrathecal baclofen therapy were assessed in the lower limbs, possible systemic or central effects cannot be completely excluded. Previous studies have suggested that botulinum toxin may exert effects beyond the local injection site through retrograde transport to the central nervous system or through indirect peripheral mechanisms influencing central motor circuits. These mechanisms have been documented in experimental and clinical studies and may potentially influence neuromuscular or reflex responses. Although the anatomical separation between injection sites and outcome assessment reduces the likelihood of a significant impact on our results, the possible systemic effects of botulinum toxin should be acknowledged as a potential confounding factor.

## 5. Conclusions

This study demonstrates that switching to baclofen 40 mg/20 mL (Neteka^®^) is safe and clinically equivalent to the conventional baclofen 10 mg/5 mL formulation in patients requiring high daily doses of intrathecal baclofen, provided that the daily dose is maintained unchanged. No clinically relevant differences were observed in spasticity control, pain management, or functional outcomes, and no formulation-related adverse events or increased end-of-dose effects occurred during the one-year follow-up period.

In contrast, a systematic reduction of the daily intrathecal baclofen dose by 10% proved to be clinically unsustainable in patients with high dose requirements and consistently required restoration of baseline dosing. This finding suggests that dose reduction strategies may not be appropriate in this patient population, likely due to a limited therapeutic margin.

Although dose reduction was not feasible, the documented long-term stability of the 40 mg/20 mL formulation provides a rationale for future investigations aimed at optimizing pump refill strategies. In particular, studies focusing on patients with lower daily intrathecal baclofen requirements may help determine whether extended refill intervals can be safely achieved without compromising clinical efficacy or safety.

## Figures and Tables

**Table 1:** Longitudinal assessment of clinical outcomes in patients with unchanged daily intrathecal baclofen dose (Group A). Data are presented as mean ± SD.

Outcome	T0	T1 (15 Days)	T2 (30 Days)	T3 (45 Days)	T4 (60 Days)	*p*-Value
MAS	2.7 ± 0.16	2.6 ± 0.23	2.5 ± 0.19	2.6 ± 0.18	2.7 ± 0.16	0.07
FIM	33.5 ± 1.64	34.3 ± 1.58	34.9 ± 2.07	33.7 ± 1.29	34.3 ± 1.44	0.19
PSFS	1.51 ± 0.13	1.51 ± 0.16	1.49 ± 0.13	1.49 ± 0.10	1.51 ± 0.10	0.92
VAS	4.53 ± 1.36	4.13 ± 1.14	4.13 ± 0.99	4.07 ± 1.02	3.93 ± 0.96	0.29
Clonus	3.6 ± 1.06	3.4 ± 0.99	3.6 ± 0.91	3.7 ± 0.88	3.5 ± 0.83	0.74

**Table 2:** Comparison of clinical outcomes at baseline, during dose reduction, and after dose restoration in patients under-going a 10% baclofen dose reduction (Group B). Data are presented as mean ± SD. * Values recorded at the time of maximal clinical worsening prior to restoration of baseline dose.

Outcome	Baseline (T0)	During Dose Reduction * (T1, 15 Days)	After Dose Restoration (T5, 75 Days)	*p*-Value
MAS	2.0 ± 0.55	3.0 ± 0.41	2.0 ± 0.51	T0 vs. T1: *p* = 0.011 T1 vs. T5: *p* = 0.017 T0 vs. T5: *p* = 1.000
FIM	33.73 ± 1.44	38.67 ± 2.77	32.67 ± 1.18	T0 vs. T1: *p* = 0.004 T1 vs. T5: *p* < 0.001 T0 vs. T5: *p* = 0.168
PSFS	1.51 ± 0.12	2.13 ± 0.52	1.49 ± 0.11	T0 vs. T1: *p* = 0.005 T1 vs. T5: *p* < 0.001 T0 vs. T5: *p* = 1.000
VAS	4.55 ± 1.36	6.53 ± 0.64	4.60 ± 0.91	T0 vs. T1: *p* = 0.005 T1 vs. T5: *p* = 0.003 T0 vs. T5: *p* = 1.000

## Data Availability

The original contributions presented in this study are included in the article. Further inquiries can be directed to the corresponding author.

## Abbreviations

The following abbreviations are used in this manuscript:

UMS	Upper Motoneuron Syndrome
ITB	Intrathecal Baclofen
MAS	Modified Ashworth Scale
PSFS	Penn Spasm Frequency Scale
FIM	Functional Independence Scale
VAS	Visual Analog Scale
FDA	Food and Drugs Administration
GABA-b	Gamma-Amino Butyric Acid
CFS	Cerebrospinal fluid
MD	Medical Doctor
